# Differential GNSS and Vision-Based Tracking to Improve Navigation Performance in Cooperative Multi-UAV Systems

**DOI:** 10.3390/s16122164

**Published:** 2016-12-17

**Authors:** Amedeo Rodi Vetrella, Giancarmine Fasano, Domenico Accardo, Antonio Moccia

**Affiliations:** Department of Industrial Engineering, University of Naples Federico II, Piazzale Tecchio 80, Naples 80125, Italy; g.fasano@unina.it (G.F.); domenico.accardo@unina.it (D.A.); antonio.moccia@unina.it (A.M.)

**Keywords:** cooperative navigation, unmanned aerial vehicles, multi-UAV Systems, differential GNSS, vision-based tracking, vision-based navigation, TRIAD method, sensor fusion, flight tests

## Abstract

Autonomous navigation of micro-UAVs is typically based on the integration of low cost Global Navigation Satellite System (GNSS) receivers and Micro-Electro-Mechanical Systems (MEMS)-based inertial and magnetic sensors to stabilize and control the flight. The resulting navigation performance in terms of position and attitude accuracy may not suffice for other mission needs, such as the ones relevant to fine sensor pointing. In this framework, this paper presents a cooperative UAV navigation algorithm that allows a chief vehicle, equipped with inertial and magnetic sensors, a Global Positioning System (GPS) receiver, and a vision system, to improve its navigation performance (in real time or in the post processing phase) exploiting formation flying deputy vehicles equipped with GPS receivers. The focus is set on outdoor environments and the key concept is to exploit differential GPS among vehicles and vision-based tracking (DGPS/Vision) to build a virtual additional navigation sensor whose information is then integrated in a sensor fusion algorithm based on an Extended Kalman Filter. The developed concept and processing architecture are described, with a focus on DGPS/Vision attitude determination algorithm. Performance assessment is carried out on the basis of both numerical simulations and flight tests. In the latter ones, navigation estimates derived from the DGPS/Vision approach are compared with those provided by the onboard autopilot system of a customized quadrotor. The analysis shows the potential of the developed approach, mainly deriving from the possibility to exploit magnetic- and inertial-independent accurate attitude information.

## 1. Introduction

In the last few years, miniaturization of flight control systems and payloads, and the availability of computationally affordable algorithms for autonomous guidance, navigation and control (GNC), have contributed to an increasing diffusion of micro-unmanned aircraft systems (micro-UAS). Besides military applications, micro-UAS can play a key role in several civil scenarios, and the attention of international top level companies and research centers has been focused on the adoption of these systems for commercial purposes [1,2] and on the paradigms for a safe and profitable access of micro-unmanned aerial vehicles (micro-UAVs) to civil airspace [3,4].

Micro-UAV navigation is typically based on the integration of low cost GNSS receivers and commercial grade Micro-Electro-Mechanical Systems (MEMS)-based inertial and magnetic sensors. An extensive review of techniques based on the integration of low cost Inertial Measurement Units (IMUs) and GNSS can be found in [5]. However, these navigation systems, only provide position accuracies of approximately 5–10 m and attitude accuracies of approximately 1°–5°, which are good enough to realize automated waypoint following, but are insufficient for most of the remote sensing or surveying applications in which fine sensor pointing is required [6,7]. Furthermore, collaborative sensing and data fusion frameworks are based on data registration as a fundamental pre-requisite, which may be directly correlated with navigation accuracy. There exist different approaches to improve UAV navigation performance.

A direct solution is to utilize high performance navigation systems. As an example, high accuracy aerial mapping systems, besides adopting dual frequency GPS receivers for accurate positioning with respect to fixed ground stations, usually exploit tactical grade IMU and/or dual GPS antenna architectures explicitly aimed at improving heading accuracy. The main disadvantages of this approach are in terms of cost and challenges related to installing dual antenna configurations on-board small UAVs. In fact, in [6] a heading accuracy of the order of 0.2°–0.5° is attained by installing the two antennas with a baseline of 1 m.

Some authors have instead followed an approach based on developing upgraded algorithmic solutions to enhance navigation performance for given low accuracy MEMS sensors. As an example in de Marina et al. [8], the Three-Axis Determination (TRIAD) algorithm [9] is used to measure the Direct Cosine Matrix (DCM) of a fixed wing aircraft, where the two reference vectors are the Earth’s magnetic field vector and gravity in North East Down (NED) coordinates, while the two observation vectors are obtained by means of magnetometers and accelerometers in the Body Reference Frame (BRF). This method is not applicable in all flight conditions, especially in presence of an external magnetic field that corrupts the magnetic measurements or when accelerations acting on the aircraft do not allow a precise identification of the gravity observation unit vector. The authors assume an attitude accuracy requirement of 1.0° on pitch and roll and 4.0° on heading, as required by industry for a fixed wing aircraft [10]. In Valenti et al. [11], attitude is obtained from the observation of the gravity and magnetic fields, where the degrading effects of magnetic disturbances on pitch and roll are mitigated separating the problem of finding the tilt and the heading quaternion with an improvement in attitude estimation. However the heading angle uncertainty is still of the order of 10°. In both cases the algorithmic improvements cannot overcome technological limitations of consumer grade IMUs.

Another approach is to integrate electro-optical sensors to detect and track natural or manmade features. Some of these vision-based techniques require the a-priori knowledge of ground control points of known appearance [12,13] in order to find homologous pairs between an on-board geotagged database and the images taken by the flying viewing system. Others estimate the egomotion of the vehicle relying only on the motion of features in consecutive images [14,15,16,17]. Moreover, several Simultaneous Localization and Mapping (SLAM) techniques have been developed [18,19] in which the vehicle build a map of the environments while simultaneously determining its location. Indeed, SLAM techniques are usually considered to limit the drift induced by inertial sensors when flying in GPS-denied and unknown environments, more than to improve navigation accuracy under nominal GPS coverage. 

Furthermore, vision-aided SLAM approaches present limits such as the necessity to detect and track natural or manmade features in a sequence of overlapping images which require a static and textured scene in good illumination conditions. This is not the case when UAS are flying over areas covered by snow, wood, sand or water e.g., day and night Search and Rescue (S&R) or natural hazards missions. Open issues to be solved remain to render the approach generally operational such as accumulated drift over time, computational complexity and data association.

The aforementioned methods exploit only one micro-UAS. However, due to single micro-UAS limits in terms of reliability, coverage and performance, multi-UAV systems have encountered increasing interest in the unmanned systems community [20,21,22,23] both for military and civil applications.

Within this framework, most of the research on cooperative navigation techniques focuses on GPS-challenging or denied environments. In Merino et al. [24] navigation in GPS-denied areas is performed acquiring overlapping images of the scene from different UAS in which at least one of them has GPS coverage. Once blob features [25] are matched among those images, it is possible to recover UAS's relative positions and consequently the absolute position of each vehicle. A similar approach has been followed by Indelman et al. [26] and Melnyk et al. [27] in which overlapping views are processed in order to evaluate relative positions in swarms. Heredia et al. [28] continued the work presented in [24] addressing the open issue of reliability, developing a Fault Detection and Identification (FDI) technique.

These Cooperative Localization (CL) techniques, in addition to the vision-based approaches drawbacks mentioned above, are affected by the need of acquiring multiple images from different platforms with an overlap that ranges from 50% up to 80% which limits the vehicles speed and requires an assigned distance between the platforms depending on the flight height and the Field of View (FOV).

This paper presents a new approach to improve the absolute navigation performance of a formation of UAVs flying in outdoor environments under nominal GPS coverage, with respect to the one achievable by integrating low cost IMUs, GNSS and magnetometers. The developed concept is to use the required formation of UAVs to build virtual navigation sensors that provide additional measurements, which are based on DGPS among flying vehicles and visual information and are not affected by magnetic and inertial disturbances. The architecture exploits cooperative GPS, as in multi-antenna attitude estimation architectures [29,30,31]. In particular, while the latter ones exploit carrier phase processing and short baselines (known by calibration) between antennas rigidly mounted on the vehicle, the new approach described in this paper is to exploit differential GPS using antennas embarked on different vehicles, where the exact geometry among them is unknown, but the line of sight between antennas can be estimated by vision sensors. 

The main innovative points are: the cooperative nature of the UAV formation is exploited to obtain drift-free navigation information; for the first time, UAV absolute attitude is estimated combining DGPS among flying vehicles and vision-based information. Indeed, the exploitation of DGPS among flying vehicles to derive attitude information is a novel concept itself. Compared with traditional navigation systems, the main advantages of our method are:

- The possibility to attain high accuracy navigation performance (e.g., sub-degree attitude measurement accuracy) without requiring high cost avionics technologies, known ground features, or textured ground surfaces.
- Reduced computational complexity.
- Independency of the navigation information from magnetic disturbances and inertial errors, also allowing better estimation of biases for these sensors [32].
- Absence of error drifts in time.

The main disadvantage is the need of keeping vehicles within the camera(s) FOV in a multi-UAV scenario. However, considering typical performance limitations of micro-UAVs, a multi-vehicle architecture could be adopted, regardless of navigation needs, in order to improve coverage and reliability. Then, the proposed approach does not require close formation control and precise relative navigation, which makes its implementation much easier. Finally, considering the cost of micro-UAV systems with consumer grade avionics, having several UAVs can be more cost effective than equipping a single vehicle with high performance navigation hardware.

The objectives of this work are as follows:

- To present a cooperative navigation architecture that is able to ensure improved navigation performance in outdoor environment, with a major focus on attitude estimation based on differential GPS and vision-based tracking. This is done in Section 2 and Section 3;
- To evaluate the achievable attitude accuracy in a numerical error analysis, pointing out the effects of DGPS/Vision measurement uncertainties and formation geometry. This is presented in Section 4;
- To compare, in flight tests, cooperative navigation output with traditional single vehicle-based data fusion implementations, thus highlighting main performance advantages of the proposed approach Section 5 and Section 6. In particular, in Section 5 a validation strategy is presented in which a ground control point is used to evaluate DGPS/Vision attitude accuracy. Experimental results are reported in Section 6.

## 2. Cooperative Navigation Architecture

As stated above, cooperation is here exploited to improve the absolute navigation performance of formation flying UAVs in outdoor environments. This is done thanks to an architecture that integrates differential GPS and relative sensing by vision (defined as “DGPS/Vision” in what follows) within a customized sensor fusion algorithm.

Considering a formation of at least two UAVs flying cooperatively, the objective is to improve the absolute navigation performance for a “chief” vehicle, equipped with inertial and magnetic sensors, a GPS receiver, and a vision system, thanks to “deputy” vehicles equipped with GPS antenna/receivers and flying in formation with the chief. On the other hand, if GPS observables are exchanged among all the vehicles, and if each vehicle is able to track at least another UAV by one or more onboard cameras, each vehicle can exploit cooperation to improve its absolute navigation performance (i.e., each vehicle can be a chief exploiting information from other deputies). The proposed cooperative navigation technique can be used either in real time or in post processing phase. In the former case, proper communication links have to be foreseen among vehicles.

In the following we assume that:

- GPS is available for all vehicles that comprise the formation.
- Each vehicle attains the same absolute positioning accuracy.

On the basis of these assumptions, the DGPS/Vision method, described in this paper, focuses on improving the UAV attitude accuracy, leaving to the sensor fusion algorithm the position and velocity improvement, as shown in [33].

**Figure 1:**
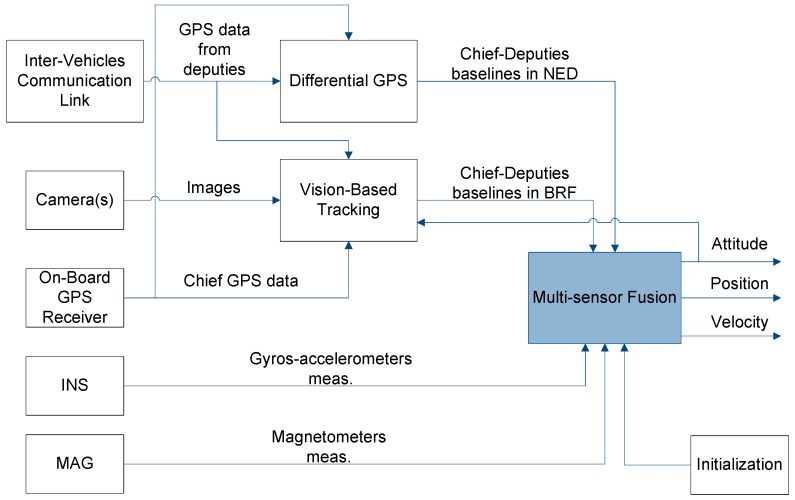
Logical architecture.

The overall cooperative navigation architecture is shown in Figure 1 where input data include: GPS measurements from the chief and the deputies; images (taken by the chief) of deputies within the FOV; inertial/magnetic data provided by the chief onboard IMU. Three processing steps are then involved:

- The vision-based tracking algorithm that allows extracting chief-to-deputies unit vectors in the BRF.
- The Differential-GPS (DGPS) block which returns chief to deputies baselines in a stabilized NED reference frame.
- A multi-sensor fusion algorithm based on an Extended Kalman Filter (EKF), which can be used to combine different information sources obtaining a more accurate and reliable navigation solution.

As it will be made clearer in the next section, depending on the processing scheme, DGPS and vision-based information can be directly integrated in the EKF, or it can be used to provide an attitude estimate that is then integrated as an additional measurement within the state estimation filter. The latter solution is the one considered in this work.

## 3. DGPS/Vision Attitude Determination Method

Vision-based tracking provides chief-deputies unit vectors in the Camera Reference Frame (CRF), which are then converted into the BRF either by only using a constant rotation matrix (accurately estimated off-line) in the case of strapdown installation, or by exploiting gimbal rotation angles in the case of gimbaled installation. 

The available GPS data, and the estimated chief attitude, can be used to cue the vision-based tracking system and individuate search windows within acquired images, improving target detection reliability and significantly reducing processing time. As an example, deputies can be tracked in the video sequences by adopting template matching approaches based on computing and maximizing the Normalized Cross Correlation (NCC) [34]. This provides an estimate of deputy centroid in pixel coordinates, which are then converted into line-of-sight (LOS) information by the intrinsic camera model.

Vision-based tracking performance for given chief/deputy platforms basically depends on the range to deputies, on environmental conditions (impacting deputy appearance, contrast and background homogeneity), and on camera(s) parameters, such as quantum efficiency and instantaneous field of view (IFOV). Moreover, camera FOV limits the maximum angular separation between deputies that can be exploited.

In order to increase the detection range performance for a given sensor, the IFOV can be reduced increasing optics focal length, and thus reducing the overall FOV and the possibility to detect widely separated deputies. The trade-off between coverage and detection range can be tackled by installing higher resolution sensors and/or multiple camera systems. 

As concerns DGPS, it can be carried out in different ways such as carrier phase differential and code-based differential processing [35]. Dual frequency carrier phase DGPS provides the most accurate relative positioning solution (cm-level error) adopting relatively expensive onboard equipments and also paying the cost of a significant computational weight [35]. Dual frequency GPS receivers are uncommon on micro UAVs, with some exceptions [36]. Indeed, a lower accuracy can be obtained even with single frequency carrier phase differential processing, provided that the integer ambiguity is solved.

The solution adopted in this work, is code-based DGPS, which requires hardware that is affordable for commercial micro UAVs, less observables to be exchanged between different vehicles (basically, only pseudoranges from common satellites in view) and much lighter processing.

A basic estimate of code-based DGPS relative positioning accuracy can be obtained multiplying typical Diluition of Precision (DOP) values by the average User Equivalent Range Error (UERE) accuracy in DGPS operating scenarios. Indeed, this is a conservative approach since pseudorange measurements correlation is increased in differential architectures. This computation leads to a typical 1-sigma accuracy of the order of 0.99 m (horizontal) and 1.86 m (vertical) [37,38,39].

While this uncertainty would correspond to a very rough angular accuracy, in the case of short baselines among rigidly mounted antennas on a single aerial platform [29,30,31], in our case a fine angular accuracy can still be attained by increasing the baselines between the chief and the deputies, thus converting position uncertainties into relatively small angular errors. 

It is also interesting to underline that within our scenarios, it is likely that the different GPS receivers use the same satellites for position fix, which leads to the possibility of a significant cancellation of common errors even by adopting position-based DGPS, i.e., by simply calculating the difference between the various position fixes.

The unit vectors (camera and DGPS) obtained by using the DGPS/Vision method, can be used to provide navigation information in different ways, such as:

- Directly integrating line of sight measurements within an EKF (which works for any number of deputies);
- TRIAD [40] (which works for two deputies);
- QUEST [41] (which works for two and more deputies).

The second and the third approach provide a straightforward attitude estimate. In this work, the focus is set on demonstrating the potential of DGPS/Vision attitude determination, thus we assume a formation with two deputies and TRIAD-based processing.

**Figure 2:**
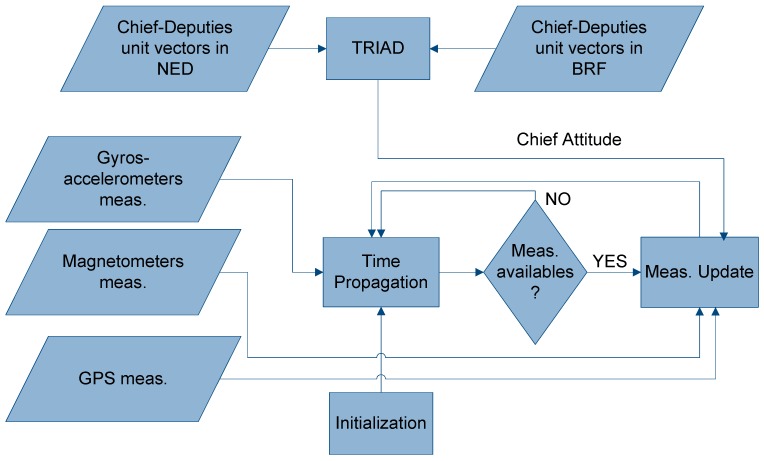
Multi-sensor fusion.

In particular, once the attitude is estimated by TRIAD, the estimate can be included as an additional measurement in a classical EKF-based aided navigation algorithm [37], which works on the basis of a prediction-correction scheme (Figure 2). The reader is referred to [42] for a detailed explanation of the navigation filter, while this paper focuses on attitude estimation aspects. 

The main advantage in using two deputies lies in the possibility to obtain direct inertial- and magnetic-independent attitude information, with a reduced computational load. Also, having two deputies improves reconfiguration capabilities of the distributed navigation sensor, as two lines-of-sight can be used to the chief advantage. The main disadvantage is the need of keeping both deputies within the FOV of chief camera(s). However, the possible challenges related to this point depend on the specifications of the adopted vision sensor(s) and the consequent trade-offs between angular accuracy and coverage.

The TRIAD algorithm [40,43,44] is an analytical method to determine the rotation matrix between two reference frames in a straightforward manner. In particular, given two nonparallel reference unit vectors ${\overset{⌢}{V}}_{1}$, ${\overset{⌢}{V}}_{2}$ in a primary reference frame and two corresponding observation unit vectors ${\overset{⌢}{W}}_{1}$, ${\overset{⌢}{W}}_{2}$ represented with respect to a secondary reference frame, TRIAD starts defining two orthonormal triads of vectors $\left\{\begin{array}{ccc} {\overset{⌢}{r}}_{1} & {\overset{⌢}{r}}_{2} & {\overset{⌢}{r}}_{3} \end{array}\right\}$ and $\left\{\begin{array}{ccc} {\overset{⌢}{o}}_{1} & {\overset{⌢}{o}}_{2} & {\overset{⌢}{o}}_{3} \end{array}\right\}$ given by:

(1) $${\overset{⌢}{r}}_{1}={\overset{⌢}{V}}_{1},\;{\overset{⌢}{r}}_{2}=\frac{{\overset{⌢}{V}}_{1}\times {\overset{⌢}{V}}_{2}}{\left|{\overset{⌢}{V}}_{1}\times {\overset{⌢}{V}}_{2}\right|},\;{\overset{⌢}{r}}_{3}={\overset{⌢}{r}}_{1}\times {\overset{⌢}{r}}_{2}$$

(2) $${\overset{⌢}{o}}_{1}={\overset{⌢}{W}}_{1},\;{\overset{⌢}{o}}_{2}=\frac{{\overset{⌢}{W}}_{1}\times {\overset{⌢}{W}}_{2}}{\left|{\overset{⌢}{W}}_{1}\times {\overset{⌢}{W}}_{2}\right|},\;{\overset{⌢}{o}}_{3}={\overset{⌢}{o}}_{1}\times {\overset{⌢}{o}}_{2}$$

and determines the unique orthogonal matrix R which converts from the primary to the secondary reference frame as follows:

(3) $$R=M_{obs}M_{ref}^{T}$$

where $M_{ref}=\left\{\begin{array}{ccc} {\overset{⌢}{r}}_{1} & {\overset{⌢}{r}}_{2} & {\overset{⌢}{r}}_{3} \end{array}\right\}$ and $M_{obs}=\left\{\begin{array}{ccc} {\overset{⌢}{o}}_{1} & {\overset{⌢}{o}}_{2} & {\overset{⌢}{o}}_{3} \end{array}\right\}$ are 3 × 3 matrices. 

As shown in Figure 2, the two vector pairs needed by the DGPS/Vision method to compute the attitude matrix are the chief-to-deputies BRF and NED (DGPS) unit vectors which are computed as explained in the following sections.

The two unit vectors in BRF are obtained starting from the pixel coordinates ($u_{i},v_{i}$) of the two deputies (i=1,2) within images acquired by the Pelican camera(s), which can be extracted by proper vision-based techniques. The normalized pixel coordinates $u_{i}^{n},v_{i}^{n}$ of the two deputies are then obtained by applying the intrinsic camera model [45,46] which takes into account the focal length, the principal point coordinates, the radial and tangential distortion coefficients, and the skew coefficient. Consequently Azimuth and Elevation and the unit vectors in CRF are then computed as follows:

(4) $$Az_{CRF_{i}}=\tan^{-1}(u_{i}^{n})$$

(5) $$El_{CRF_{i}}=\tan^{-1}(-v_{i}^{n}\cos(Az_{i}))$$

(6) $${\hat{r}}_{i}^{CRF}=\left[\begin{array}{c} {\hat{r}}_{i,1}\\ {\hat{r}}_{i,2}\\ {\hat{r}}_{i,3} \end{array}\right]=\left[\begin{array}{c} \cos(El_{CRF_{i}})\cos(Az_{CRF_{i}})\\ \cos(El_{CRF_{i}})\sin(Az_{CRF_{i}})\\ -\sin(El_{CRF_{i}}) \end{array}\right]$$

where ${\hat{r}}_{i}^{CRF}$ is the unit vector of components (${\hat{r}}_{i,1}$, ${\hat{r}}_{i,2}$, ${\hat{r}}_{i,3}$) in CRF. The unit vectors in BRF, to be used as reference vectors within the TRIAD algorithm, are given by:

(7) $${\hat{r}}_{i}^{BRF}=R_{CRF\to BRF}{\hat{r}}_{i}^{CRF}$$

where $R_{CRF\to BRF}$ is the constant rotation matrix from CRF to BRF and ${\hat{r}}_{i}^{BRF}$ is the unit vector in BRF.

The other two vectors needed to apply the TRIAD algorithm are the chief-to-deputies NED unit vectors. In this work, these vectors are obtained by adopting a Double Difference (DD) code-based DGPS solution [35], which offers significant advantages due to the cancellation of receiver and satellite clock biases, as well as most of the ionospheric and tropospheric propagation delays. To this end, it is assumed that the chief and the two deputies are in view of the same n satellites and consequently their pseudorange measurements are available which allow to calculate single and DD observables. In particular, considering the chief and the *i*-th deputy, and two GPS satellites, one of which named pivot, DD observables are obtained as follows

(8) $$PR_{ci}^{pk}=(PR_{i}^{k}-PR_{i}^{p})-(PR_{c}^{k}-PR_{c}^{p})$$

where the superscript p refers to the pivot GPS satellite, which is chosen to be the one with the highest elevation, k refers to the generic satellite (k=1,...,n−1), the subscripts c and i represent the chief and the *i*-th deputy vehicle GPS receiver respectively, $PR_{i}^{k}$ stands for the pseudorange estimated by the *i*-th receiver with respect to the *k*-th satellite, and a similar interpretation holds for the other estimated pseudoranges. 

The DD observation model is a non linear function of the baseline $\Delta {\underline{r}}_{i}^{ECEF}$ between the chief and *i*-th deputy in the Earth Centered Earth Fixed (ECEF) reference frame as shown in the following equation:

(9) $$\begin{array}{c} PR_{ci}^{pk}=\rho_{ci}^{pk}+\nu_{ci}^{pk}=\\ \left\|{\underline{R}}_{k}-\left({\underline{r}}_{c}+\Delta {\underline{r}}_{i}^{ECEF}\right)\right\|-\left\|{\underline{R}}_{k}-{\underline{r}}_{c}\right\|-\left\|{\underline{R}}_{p}-\left({\underline{r}}_{c}+\Delta {\underline{r}}_{i}^{ECEF}\right)\right\|+\left\|{\underline{R}}_{p}-{\underline{r}}_{c}\right\|+\nu_{ci}^{pk} \end{array}$$

where $\rho_{ci}^{pk}$ represents the DD between the true pseudoranges, ${\underline{r}}_{c}$ is the chief ECEF position, ${\underline{R}}_{p}$ and ${\underline{R}}_{k}$ are the pivot and *k*-th satellites ECEF positions and $\nu_{ci}^{pk}$ are the non-common mode pseudorange errors. The problem of finding $\Delta {\underline{r}}_{i}^{ECEF}$ is solved applying a recursive least square estimation method based on the linearization of the DD observation model [35].

The *i*-th baseline $\Delta {\underline{r}}_{i}^{NED}$ in the NED reference frame, with origin in the chief center of mass (usually defined as “navigation frame” [37]), is then given by:

(10) $$\Delta {\underline{r}}_{i}^{NED}=R_{ECEF\to NED}\Delta {\underline{r}}_{i}^{ECEF}$$

where $R_{ECEF\to NED}$ is the rotation matrix from the ECEF to the navigation frame that depends on the chief longitude λ and geodetic latitude μ.

(11) $$R_{ECEF\to NED}=\left[\begin{array}{ccc} -\sin\mu \cos\lambda & -\sin\mu \sin\lambda & \cos\mu \\ -\sin\lambda & \cos\lambda & 0\\ -\cos\mu \cos\lambda & -\cos\mu \sin\lambda & -\sin\mu \end{array}\right]$$

Of course, the accuracy of the resulting attitude estimate depends on several factors such as DGPS and vision-based tracking errors, formation geometry, and chief vehicle attitude. These aspects are analyzed in the following section.

For the case of n deputies (i=1,…,n), n unit vectors can be computed in BRF and NED by applying Equations (4)–(7) and (8)–(11), respectively. In this case, the optimal solution is given by the QUEST algorithm [41] which minimizes a quadratic cost function involving an arbitrary number of vector measurements made in BRF and NED.

## 4. Error Analysis

In order to analyze the performance of the DGPS/Vision sensor, a numerical approach has been followed. In particular, for a given chief attitude and formation geometry, DGPS and optical measurements are simulated by random extractions, and the resulting attitude measurement error is analyzed with statistical tools.

Indeed, an analytical solution exists which allows estimation of TRIAD attitude error covariance matrix [40] as a function of formation geometry and line-of-sight uncertainties. The basic assumptions underlying this derivation are that errors must to first order lie in the plane perpendicular to the respective unit vector, and have an axially symmetric distribution about it.

The second assumption clearly does not hold in the architecture considered in this paper, due to the difference in GPS performance between the horizontal plane and the vertical direction [37,38,39]. Worst case or averaging approaches in using TRIAD covariance matrix equation are clearly sub-optimal and can produce over or under-conservative results. 

Regarding the numerical simulation architecture, the usual 321 sequence of Euler angles (heading, pitch, and roll), and the case of chief null attitude angles are assumed. In general, each deputy is instantaneously located at given azimuth and elevation angles (*ε_i_* and *μ_i_*) with respect to the body reference frame of the chief, and at a different range (*L_i_*). In order to underline the main effects on measurement accuracy, formation geometry can be conveniently described in terms of azimuth center $\varepsilon_{c}$ and azimuth separation Δε between the deputies:

(12) $$\varepsilon_{c}=\frac{\varepsilon_{1}+\varepsilon_{2}}{2}$$

(13) $$\Delta \varepsilon =\varepsilon_{1}-\varepsilon_{2}$$

**Figure 3:**
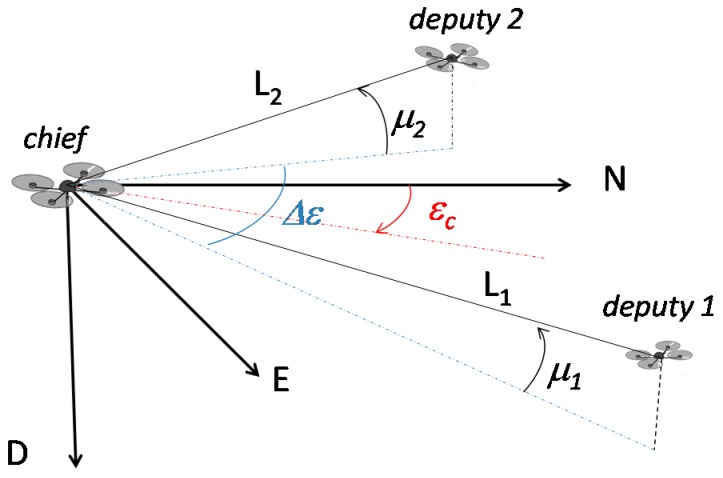
Formation geometry parameters.

Formation geometry is depicted in Figure 3.

EO-based azimuth and elevation errors are simulated as zero-mean gaussian noises with standard deviation equal to 0.05°, which is consistent with typical IFOV values of sensors commonly found onboard micro-UAVs. For the sake of simplicity, the case of equal range and elevation is considered for the two deputies. Then, three cases are analyzed:

- Case 1: horizontal geometry with azimuth center at 0°;
- Case 2: “tilted” geometry with azimuth center at 0°;
- Case 3: horizontal formation with azimuth center at 45°.

For each case, a variable angular separation between deputies is considered. The three cases are summarized in Table 1.

**Figure 4:**
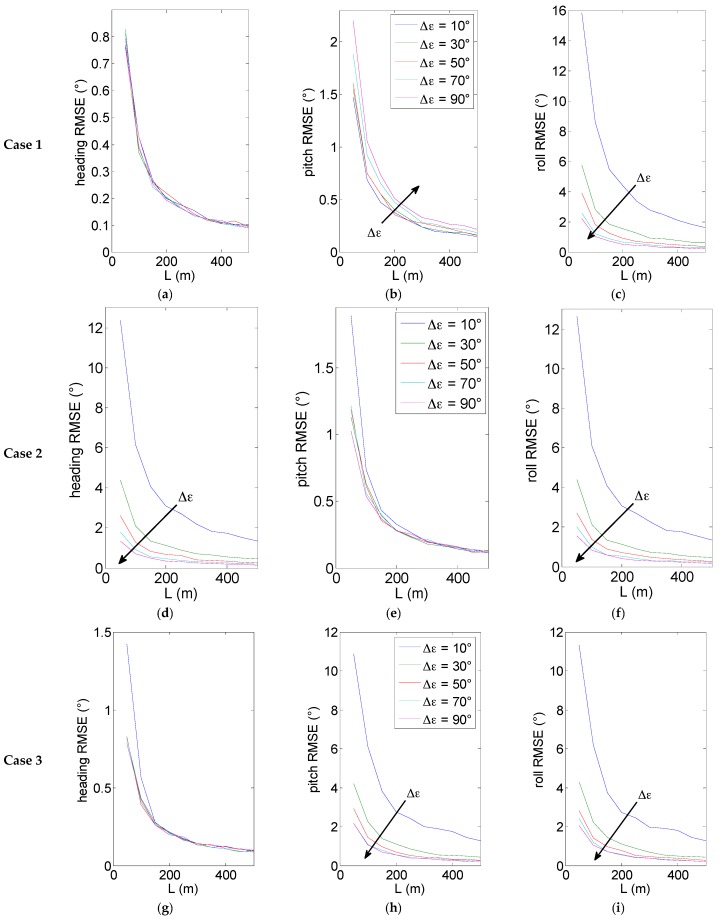
Attitude root mean square errors (RMSE) as a function of baseline L, for different values of angular separation. Case 1 (**a**–**c**) heading pitch and roll RMSE for *μ = **ε_c_** =* 0*°*; Case 2 (**d**–**f**) heading pitch and roll RMSE for *μ* = 45° and ***ε*_c_** = 0°; Case 3 (**g**–**i**) heading pitch and roll RMSE for *μ* = 0° and ***ε*_c_** = 45°.

Results based on 200 numerical simulations are shown in Figure 4 for the three considered cases. Figure 4 offers several points of discussion.

The above diagrams show as expected that increasing the baseline improves the attitude estimation accuracy, with an error dependency on 1/L. This is mainly related to how angular differential GPS uncertainty decreases by increasing the baseline between the antennas. As already stated above, this advantage must be traded-off against the performance of vision-based tracking, given the decreasing dimensions in pixels of the deputies. As an example, an instantaneous field of view of 0.05° corresponds at a distance of 100 m to a geometric resolution of 9 cm. Consequently, a longer baseline requires an improvement of the geometric resolution of the vision sensors which can be attained by decreasing the FOV or installing a multi-camera system on the chief. Furthermore, differences among attitude angles are due to the different contributions of horizontal and vertical DGPS uncertainties.

As regards the relation between formation geometry and attitude accuracy, it is clear that in all cases the impact of line of sight uncertainties on attitude determination errors mainly depends on the angle between unit vectors and the considered chief body axes. 

In Case 1 (Figure 4a), heading error does not depend on ∆ε, since in all cases unit vectors are normal to the third axis of the BRF. In the considered formation geometry the heading uncertainty basically depends only on the horizontal GPS error and thus exhibits the finest accuracy: the uncertainty is always well below 1°, and fast approaches 0.1° for increasing baselines. This is a very useful result coming out from the proposed DGPS/Vision sensor to be pointed out considering typical high uncertainties in estimating magnetic heading on board small and micro UAVs and the consequent interest in high cost compact navigation systems (e.g., based on high cost IMUs and/or dual antenna GNSS) [6,7].

As regards the other angles (Figure 4b,c), for increasing angular separation pitch accuracy decreases while the roll accuracy increases. These effects are due to the varying angles between unit vectors and the coordinate axes.

In particular, effects on pitch are limited for the considered range of angular separations, while a much larger effect is present for roll. These effects would further increase if the angular separation approached 180°. The considered geometry, especially for small angular separation, is optimal for pitch estimation. However, the final pitch accuracy does not reach the heading one due to the fact that it (only) depends on the (larger) GPS vertical error.

Case 2 (Figure 4) highlights the effects of non-horizontal formation geometry. Compared with Case 1, it is clear how heading accuracy decreases due to the non-optimal observation geometry (smaller angles between unit vectors and third body axis). Instead, pitch and roll estimates are positively influenced by the non-null elevation angle. In particular, pitch estimate takes advantage from depending not only on the vertical GPS error, but also on the smaller horizontal component. Instead, benefits for roll mainly derive from the increased angles among unit vectors and the first body axis. Deputy separation does not influence significantly pitch accuracy, while its increase is beneficial for both heading and roll angles. In this case, among the three angles, pitch has the highest accuracy.

As regards Case 3 (Figure 4), this asymmetric geometry does not impact significantly heading accuracy, which is very similar to Case 1 and thus very weakly influenced by deputy separation. Instead, both pitch and roll errors are positively impacted by increasing separation, due to the fact that one of the unit vectors tends to be normal to the first or second body axis, as ∆ε approaches 90°. In fact, the latter geometry represents an optimal compromise in terms of accuracy for all the three angles, which represents a well known result exploited in designing single vehicle multi-antenna GPS configurations [29,30,31]. For example, at 100 m baseline we have an error standard deviation of 0.4° for heading, and of 1° for roll and pitch.

In summary, these budgets show that even the relatively coarse code-based DGPS processing is very promising in terms of attitude estimation, when combined with vision-based sensing. In particular, horizontal geometries provide a very fine heading accuracy even at relatively short baselines, while asymmetries can be useful to find a balance between roll and pitch errors. In practical cases, the choice of formation geometries will depend on the requirements for final navigation performance (sensor fusion output) and on eventual constraints deriving from the flight environment. Also, formation reconfiguration strategies can be envisaged to keep required levels of attitude estimation performance.

## 5. Testing and Validation Strategy

In order to evaluate the performance of the novel DGPS/Vision algorithm, experimental tests are described where the chief vehicle is a customized quadrotor, and two ground-based GPS antennas/receivers are used as surrogate deputies.

### 5.1. Experimental Setup

**Figure 5:**
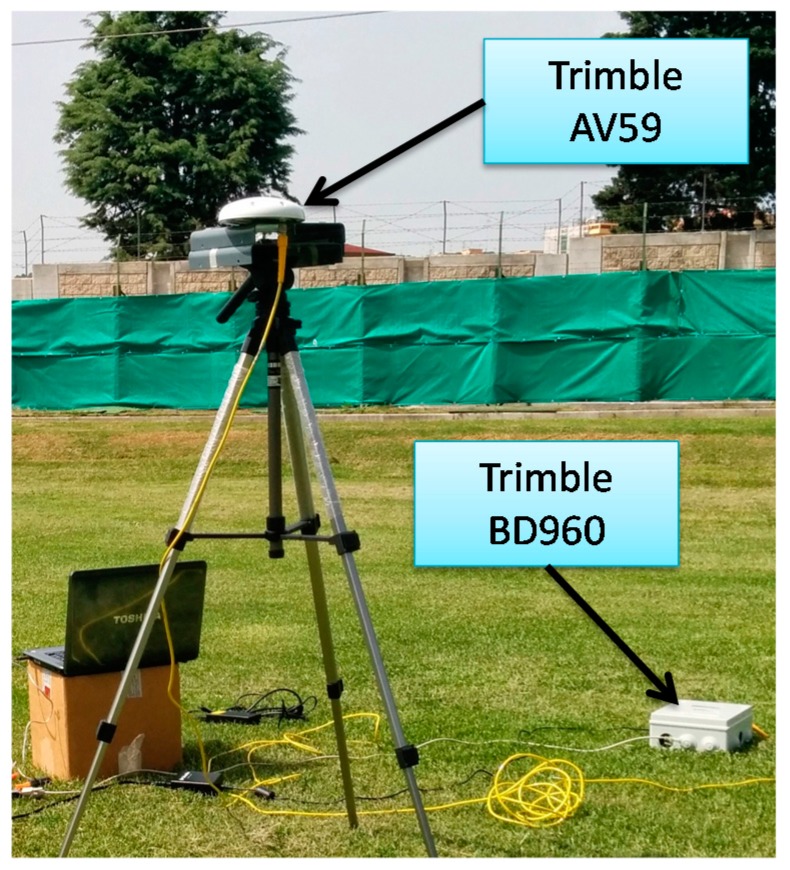
Ground antennas/receivers used as deputy vehicles.

**Figure 6:**
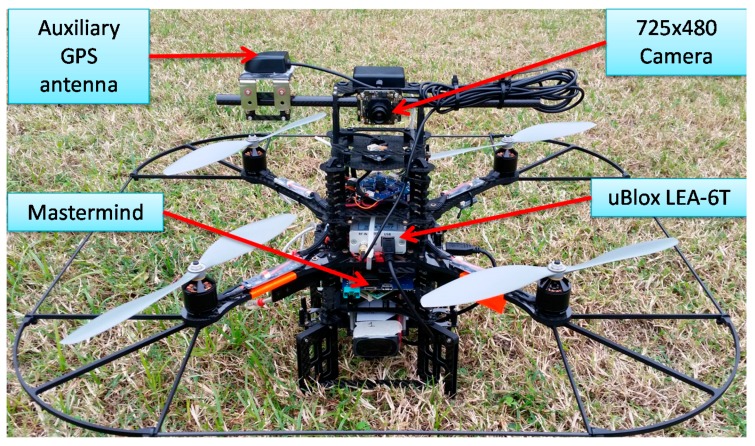
Chief vehicle (Customized Ascending Technologies^TM^ Pelican).

Tests have been conducted by using two surrogate deputies, consisting in two ground stations equipped with an AV59 antenna (Trimble^TM^, Sunnyvale, CA, USA) and BD960 receiver (Trimble^TM^, Sunnyvale, CA, USA, Figure 5) observed by a Pelican quadrotor (Figure 6) (Ascending Technologies^TM^, Krailling, Germany) [47] that plays the role of chief.

In particular, the Pelican quadrotor, besides being equipped with a controller, a set of onboard sensors (Table 2) and an onboard computer (AscTec^TM^ Mastermind, Ascending Technologies^TM^, Krailling, Germany), has been customized with a miniaturized electro optical sensor (BlueFox^TM^ MLC200wC, Matrix Vision, Brescia, Italy) and an additional uBlox^TM^ GPS Receiver (LEA 6T, uBlox^TM^, Thalwil, Switzerland) which provides raw measurements that are used in the DGPS processing.

The additional GPS receiver and the optical sensor have been connected to the Mastermind computer via a USB link, while other raw and calibrated/processed sensor data are read using the UART connection between Mastermind and autopilot. The acquisition software that runs on the Mastermind has been coded in C++ and gathers all the necessary data with an accurate time-tag based on GPS time and the CPU clock. In particular, IMU data are acquired with the aid of the Asctec Communication Interface (ACI) at a frequency of 100 Hz while images and GPS raw data are gathered simultaneously at a frequency of 1 Hz.

This setup has been used to validate the presented DGPS/Vision attitude determination approach with particular emphasis on estimation of heading angle. 

### 5.2. Pointing/Attitude Accuracy Evaluation Strategy

Given the sub-degree attitude determination accuracy obtained by the DGPS/Vision method for the baselines experimented during flight tests, in particular for the heading angle, it is nontrivial to find a reference measurement that provides a ground truth of better accuracy level. In fact, this level of accuracy can be reached installing a tactical grade IMU on board the Pelican, or a dual antenna navigation system [6,7], or using very accurately geoferenced ground control points. Besides the cost, dual antenna navigation systems have a significant limit related to the necessity to install the two antennas with a sufficiently large baseline (1 m or more), which is hard to obtain onboard the Pelican.

In this work, the DGPS/Vision attitude accuracy has been evaluated by identifying on an open source 1:1000 georeferenced map [48] (planar error of the order of 25 to 50 cm) the position of a Ground Control Point (GCP) which is visible in the acquired images. 

The idea is to compute azimuth and elevation in the navigation frame of an identifiable GCP, to be used as reference measurements to evaluate the pointing accuracy.

**Figure 7:**
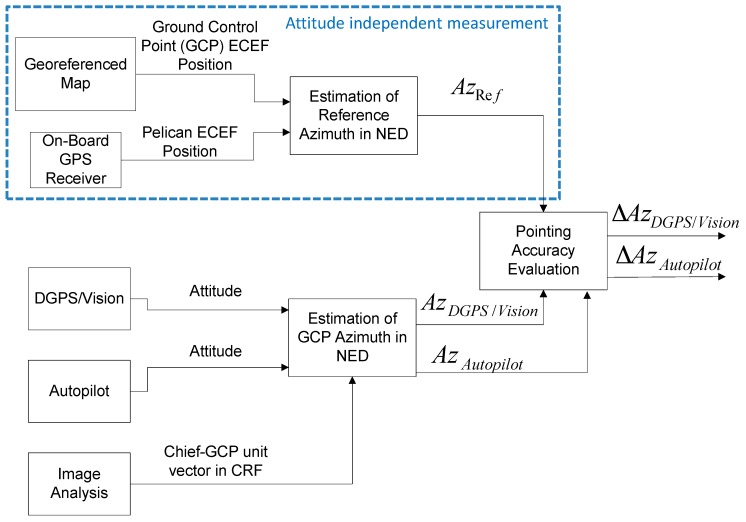
Pointing accuracy logical scheme.

The logical scheme of the pointing accuracy analysis is shown in Figure 7 where the main processing steps are highlighted. As mentioned above, the focus is on heading performance, as a consequence only the processing strategy and results concerning azimuth in NED will be further analyzed showing that azimuth accuracy represents a good benchmark to evaluate and compare heading performance. 

The (attitude independent) reference measurement is obtained starting from the ECEF relative position vector $\Delta {\underline{r}}^{ECEF}$ between the Pelican and the GCP:

(14) $$\Delta {\underline{r}}^{ECEF}=\left[\begin{array}{c} x_{GCP}-x_{Pelican}\\ y_{GCP}-y_{Pelican}\\ z_{GCP}-z_{Pelican} \end{array}\right]=\left[\begin{array}{c} \Delta x\\ \Delta y\\ \Delta z \end{array}\right]$$

where $x_{GCP}$, $y_{GCP}$ and $z_{GCP}$ are the ECEF coordinates of the GCP (provided by the map) and $x_{Pelican}$, $y_{Pelican}$ and $z_{Pelican}$ are the Pelican ECEF coordinates given by the on-board GPS receiver. $\Delta {\underline{r}}^{ECEF}$ is then converted in the NED reference frame as follows

(15) $$\Delta {\underline{r}}^{NED}=R_{ECEF\to NED}\Delta {\underline{r}}^{ECEF}$$

Once $\Delta {\underline{r}}^{NED}$ is obtained, the reference Azimuth AzRef in NED is given by

(16) AzRef=tan−1(ΔyNEDΔxNED)+kπ,{k=0 if ΔxNED>0k=1 if ΔxNED<0

where $\Delta x^{NED}$ and $\Delta y^{NED}$ are the NED-referenced relative position vector components.

**Figure 8:**
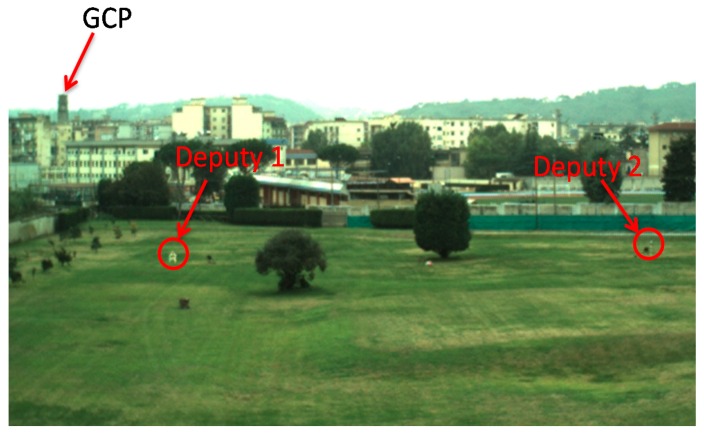
Example of flight image showing the observation geometry.

AzRef is used as a reference to evaluate the accuracy of the DGPS/Vision and Autopilot heading. To this end, the GCP (Figure 8) is identified on each image to compute the Pelican-to-GCP Line of Sight (LOS) in the CRF ${\hat{r}}^{CRF}$ and then in BRF ${\hat{r}}^{BRF}$ according to Equation (7). ${\hat{r}}^{BRF}$ is then transformed in two unit vectors, one, ${\hat{r}}_{DGPS/Vision}^{NED}$, applying the attitude matrix $R_{BRF\to NED}^{DGPS/Vision}$ computed using the DGPS/Vision method, and the other, ${\hat{r}}_{Autopilot}^{NED}$, applying the attitude matrix $R_{BRF\to NED}^{Autopilot}$ computed by the Autopilot:

(17) $${\hat{r}}_{DGPS/Vision}^{NED}=R_{BRF\to NED}^{DGPS/Vision}{\hat{r}}^{BRF};\;{\hat{r}}_{Autopilot}^{NED}=R_{BRF\to NED}^{Autopilot}{\hat{r}}^{BRF}$$

Consequently, the two azimuth errors are

(18) ΔAzDGPS/Vision=AzDGPS/Vision−AzRef, ΔAzAutopilot=AzAutopilot−AzRef

where

(19) $$Az_{DGPS/Vision}=\tan^{-1}(\frac{{\hat{r}}_{DGPS/Vision,2}^{NED}}{{\hat{r}}_{DGPS/Vision,1}^{NED}}),\;Az_{Autopilot}=\tan^{-1}(\frac{{\hat{r}}_{Autopilot,2}^{NED}}{{\hat{r}}_{Autopilot,1}^{NED}}),$$

are the estimates of the GCP azimuth in NED ($Az_{Autopilot}$, $Az_{DGPS/Vision}$) and [${\hat{r}}_{DGPS/Vision,1}^{NED}$, ${\hat{r}}_{DGPS/Vision,2}^{NED}$] and [${\hat{r}}_{Autopilot,1}^{NED}$, ${\hat{r}}_{Autopilot,2}^{NED}$] are the NED-referenced unit vector components related to DGPS/Vision and autopilot, respectively. 

Two important factors for effective application of the proposed accuracy evaluation strategy regard the uncertainty of the reference azimuth measurement given by Equation (16), and the relation between the uncertainties on attitude measurements and the azimuth angles computed in Equation (19).

As regards the first point, the georeferenced map has a planar sub-metric accuracy, while the error on Pelican positioning depends on horizontal accuracy of standalone GPS. Due to the large distance from the GCP (about 600 m), the linear uncertainty of the baseline is converted into a relatively small angular error. For the sake of concreteness, if one assumes 6 m of horizontal relative positioning error, the worst case uncertainty on the reference azimuth measurement (i.e., error vector normal to the Pelican-GCP line of sight) is given by:

(20) $$\tan^{-1}(\frac{6}{600})\approx 0.01\;\mathrm{rad}=0.57{}^{\circ}$$

As concerns the relation between azimuth and heading, it is intuitive that for small roll and pitch angles (as it indeed happens in the considered flight tests), azimuth accuracy depends primarily on heading measurement performance, with very little effect produced by the other errors. This can be demonstrated analytically by deriving a first order error budget.

In particular, given Equation (19) it is possible, both for DGPS/Vision and Autopilot, to express the azimuth uncertainty as

(21) $$\sigma_{Az}^{2}≅{\left(\frac{\partial Az}{\partial \psi }\right)}^{2}\sigma_{\psi }^{2}+{\left(\frac{\partial Az}{\partial \vartheta }\right)}^{2}\sigma_{\vartheta }^{2}+{\left(\frac{\partial Az}{\partial \varphi }\right)}^{2}\sigma_{\varphi }^{2}+{\left(\frac{\partial Az}{\partial Az_{CRF}}\right)}^{2}\sigma_{Az_{CRF}}^{2}+{\left(\frac{\partial Az}{\partial El_{CRF}}\right)}^{2}\sigma_{El_{CRF}}^{2}$$

where ψ, ϑ and φ are heading, pitch, and roll, respectively, while $Az_{CRF}$ and $El_{CRF}$ are the GCP azimuth and elevation angles computed in the camera reference frame. The squared derivatives can be computed analytically starting from Equation (19). They measure the sensitivity of azimuth uncertainty on the input errors, and depend themselves on ψ, ϑ, φ, $Az_{CRF}$, and $El_{CRF}$.

For the sake of concreteness in view of the analyzed experiment, assuming CRF coincident with BRF, null attitude angles, $Az_{CRF}=-20{}^{\circ}$, and $El_{CRF}=5{}^{\circ}$, one gets

(22) $${\left(\frac{\partial Az}{\partial \psi }\right)}^{2}≅1,{\left(\frac{\partial Az}{\partial \vartheta }\right)}^{2}≅9\cdot 10^{-4},{\left(\frac{\partial Az}{\partial \varphi }\right)}^{2}≅7\cdot 10^{-3},{\left(\frac{\partial Az}{\partial Az_{CRF}}\right)}^{2}≅1,{\left(\frac{\partial Az}{\partial El_{CRF}}\right)}^{2}≅0$$

which shows that the main contributions to azimuth pointing error are the uncertainties on ψ and $Az_{CRF}$. 

Even degree-level errors on roll and pitch have a limited contribution, since they are strongly attenuated. On the other hand, since uncertainties in $Az_{CRF}$ and $El_{CRF}$ are related to the camera IFOV and thus of the order of 0.05° in the considered case, the final uncertainty on azimuth pointing is given by a small amplification of the heading one.

As an example, assuming the uncertainties obtained in case 1 of the error analysis approach $\sigma_{\psi }=0.35{}^{\circ},\sigma_{\vartheta }=0.7{}^{\circ},\sigma_{\varphi }=3.5{}^{\circ}$, and considering $\sigma_{Az_{CRF}}=0.05{}^{\circ},\sigma_{El_{CRF}}=0.05{}^{\circ}$, one gets $\sigma_{Az}≅0.46{}^{\circ}$. This shows that azimuth accuracy evaluation represents a good benchmark to evaluate and compare heading estimation performance.

### 5.3. Flight Tests

**Figure 9:**
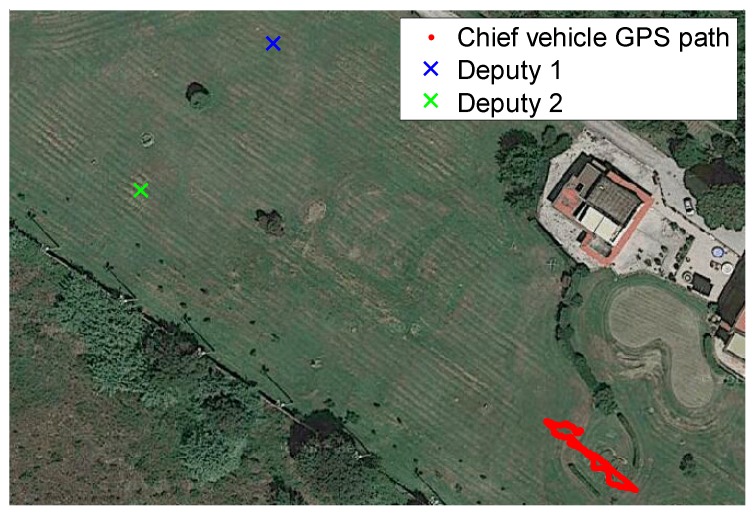
Formation geometry Test 1.

**Figure 10:**
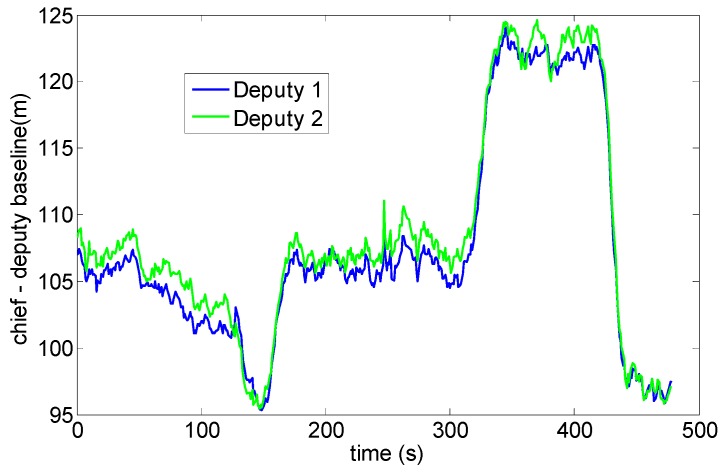
Chief-deputies baselines Test 1.

**Figure 11:**
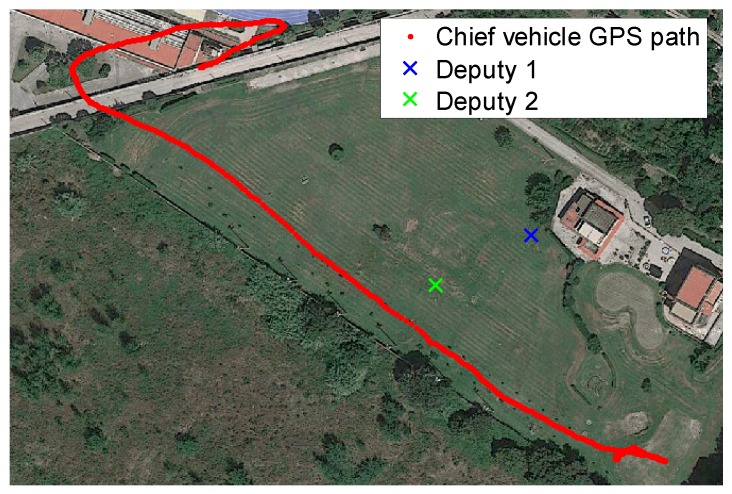
Formation geometry Test 2.

**Figure 12:**
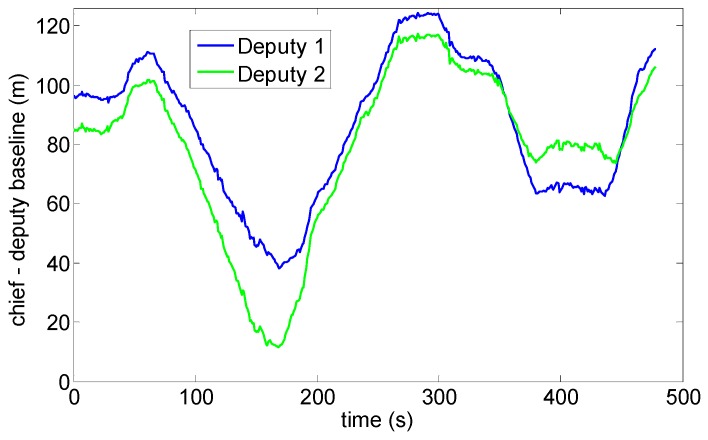
Chief-deputies baselines Test 2.

Experimental tests have been carried out in an outdoor area that allows baselines among chief and deputies of the order of 100 m. As noted above, this is necessary to reduce the DGPS angular error and thus to improve attitude determination uncertainty. Tests have been designed to compare DGPS/Vision estimates with heading measurements based on the onboard magnetometers and the output of the real time data fusion algorithm running on Pelican autopilot, based on a filter which combines accelerometers, gyroscopes and magnetometers. In particular, among the flights that have been conducted, two tests have been chosen as representative of attitude dynamics and varying formation geometry, as follows:

- Test 1: an almost constant horizontal formation geometry (Figure 9) has been kept, with a baseline between the two deputies of the order of 40 m, and the chief at a distance slightly larger than 100 m from the two ground antennas (Figure 10). A number of attitude maneuvers has been commanded, including four 360° heading rotations and 1 Hz heading oscillation with about 30° amplitude. This test has been selected to point out the different levels of robustness of onboard fusion and DGPS/Vision with respect to flight dynamics history.
- Test 2: the chief vehicle has been commanded to fly along a path of about 200 m (Figure 11), thus generating a significant change of formation geometry in NED coordinates. The two ground antennas have been positioned in order to provide a baseline of about 100 m with respect to the chief vehicle at the starting and end point of the flight path (Figure 12). This test has been selected to point out the effects on the onboard filter and on DGPS/Vision estimates of both flight dynamics history and magnetic effects.

## 6. Experimental Results

During the experimental tests, the (forward-looking) camera embarked on the chief vehicle has acquired images of the two deputy antennas while measurements from GPS receivers and other onboard sensors have also been gathered. In particular, attitude measurements obtained from the filter running on the autopilot have been stored, while attitude estimates based on differential GPS and vision have been obtained by off-line processing. 

**Figure 13:**
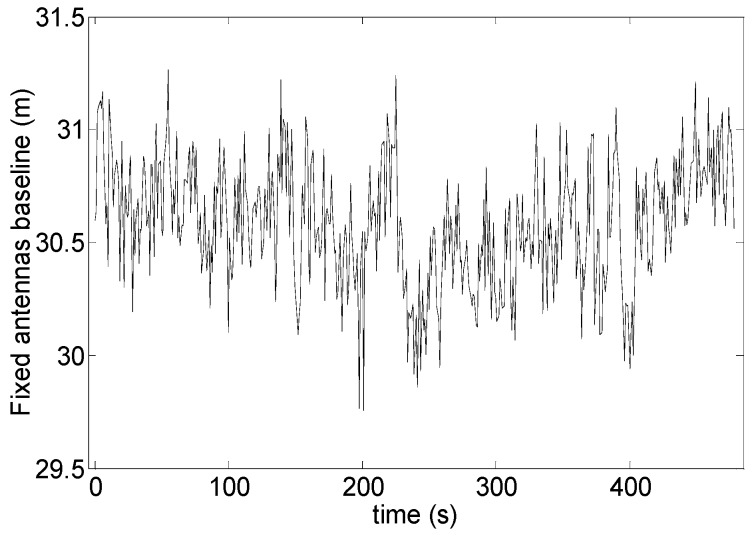
Fixed antennas baseline (DGPS) as a function of time.

Stability and noise properties of the DGPS solution can be verified by analyzing the baseline estimated between static ground antennas. This is shown in Figure 13 that regards test 1. It can be seen that the estimated baseline exhibits sub-meter oscillations, which is consistent with the error analysis presented in Section 4. 

Experimental data have been acquired in both static and dynamic conditions. Results from a static test are described in Subsection 6.1, while Subsection 6.2, Subsection 6.3, Subsection 6.4 and Subsection 6.5 report results from the previously presented flight tests.

### 6.1. Static Test

Static data acquisitions have been conducted mainly to verify the precision properties of the DGPS/Vision solution. During this test the chief vehicle has been positioned and held stationary on the ground in an almost horizontal formation geometry keeping the two ground antennas in the camera FOV at a distance slightly larger than 100 m. 

**Figure 14:**
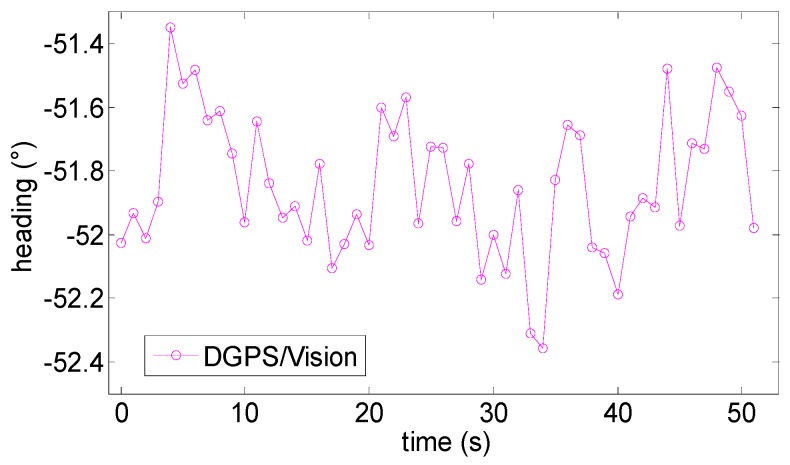
Heading angle as estimated by the DGPS/Vision as a function of time.

Static results have confirmed the drift-free behavior of DGPS/Vision measurements, while also showing a standard deviation consistent with formation geometries considered in Case 1 in the numerical simulation error budget (0.7° pitch, 3.5° roll and 0.35° heading). Indeed, the experimented attitude measurement noise has been a little smaller than numerical predictions, in fact, the heading estimated by DGPS/Vision (Figure 14) has a standard deviation of about 0.23°.

### 6.2. Heading—Test 1

Test 1 allows comparing DGPS/Vision and magnetometers/autopilot attitude estimates from the point of view of sensitivity to attitude dynamics. 

**Figure 15:**
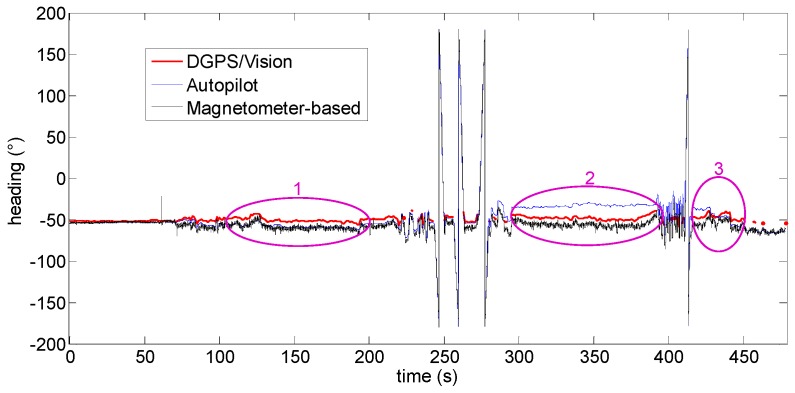
Heading angle as a function of the flight time (Test 1).

Figure 15 shows the heading angle during the flight where DGPS/Vision attitude estimates are available for most of the considered time interval. Some DGPS/Vision isolated losses are produced by the impossibility to detect both antennas within images, due to the observation geometry, the limited FOV of the camera and the maneuvers executed during the tests.

In the following, to get a clearer insight into the DGPS/Vision performance and robustness, it is worthwhile to focus the attention on three flight segments, as indicated in Figure 15. In fact, while DGPS/Vision measurements are independent from magnetic and inertial information, the Pelican data fusion shows significant limits particularly at the end of the heading rotation maneuvers. This seems due to different weights which probably the Pelican data fusion algorithm applies to the input data coming from the gyroscopes, the accelerometers and the magnetometer. 

**Figure 16:**
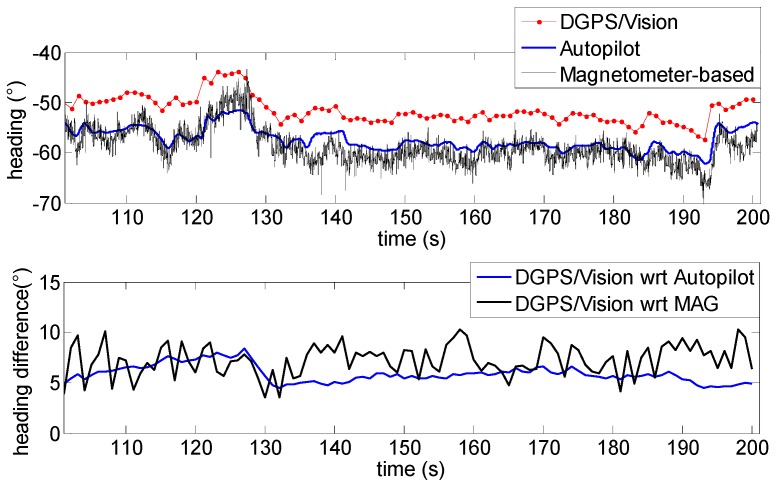
Heading angle as a function of time (**Top**), differences of DGPS/Vision with respect to autopilot and magnetometers (**Bottom**) during the first flight segment (Test 1).

In Figure 16 the low frequency profile of the heading angle during the first time interval does not introduce any significant disturbance and consequently the pelican data fusion algorithm follows quite well the heading given by the MEMS magnetometer. 

The first flight segment lies before the heading rotation maneuvers (Figure 15). Within this time interval, the difference of about 5.9° (Table 3) between DGPS/Vision and autopilot heading is almost constant, mainly due to magnetic biases. In addition, Figure 16 shows a good consistency between magnetometer-based and autopilot estimates (Table 3). 

**Figure 17:**
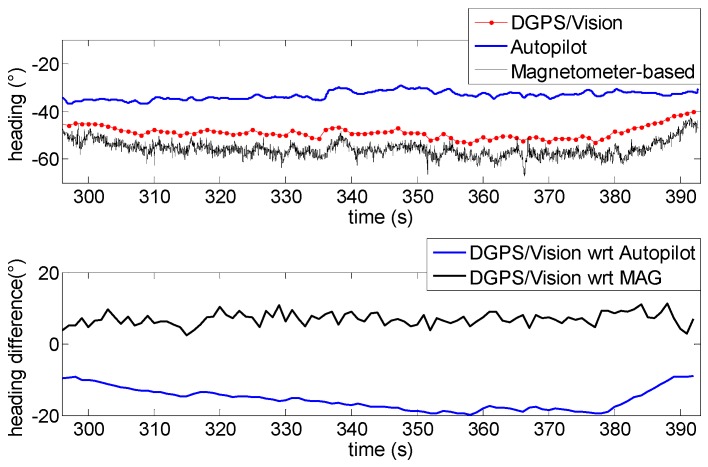
Heading angle as a function of time (**Top**), differences of DGPS/Vision with respect to autopilot and magnetometers (**Bottom**) during the second flight segment (Test 1).

The situation changes completely after the 360° heading rotations (Figure 17). While the offset between DGPS/Vision and magnetometer-based heading is very similar to the first flight segment, a significant drift of about 15° (Table 3) is generated with respect to the autopilot solution, which is mostly due to the coarse gyroscopes accuracy and the consequent data fusion limits in tracking the actual vehicle dynamics. The difference between DGPS/Vision and autopilot heading achieves a maximum value of about 20°, while a significant offset is also generated between magnetometer-based heading and autopilot heading (about 22°).

**Figure 18:**
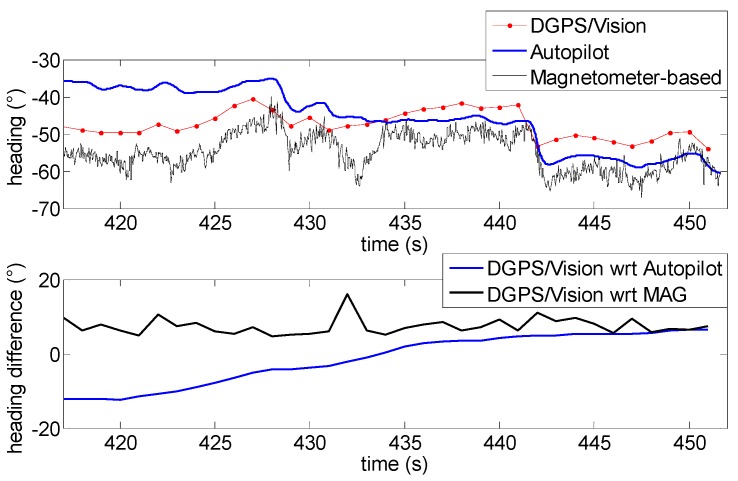
Heading angle as a function of time (**Top**), differences of DGPS/Vision with respect to autopilot and magnetometers (**Bottom**) during the third flight segment (Test 1).

As shown in Table 3, only after several tens of seconds, and further maneuvers (third flight segment, Figure 18), the autopilot solution recover the offset with respect to DGPS/Vision and magnetometer-based heading, thus achieving a final performance level that resembles the one experimented in the first flight segment. 

During all the flight test, the difference, of about 7° (Table 3), between DGPS/Vision and magnetometer-based heading does not show significant variations. 

In summary, compared with classical attitude determination techniques, this test confirms the potential of DGPS/Vision to provide small noise measurements which are completely independent from attitude dynamics history.

### 6.3. Heading—Test 2

During the second test, the Pelican has been commanded to fly along a relatively long path, covering the whole test field and generating a heading change of almost 180°. Due to the necessity to keep the antennas within camera FOV, chief motion actually combines a forward-lateral-backward translation and a very slow heading rotation. The changing aircraft orientation within the Earth magnetic field allows pointing out DGPS/Vision potential with respect to both inertial and magnetic effects.

**Figure 19:**
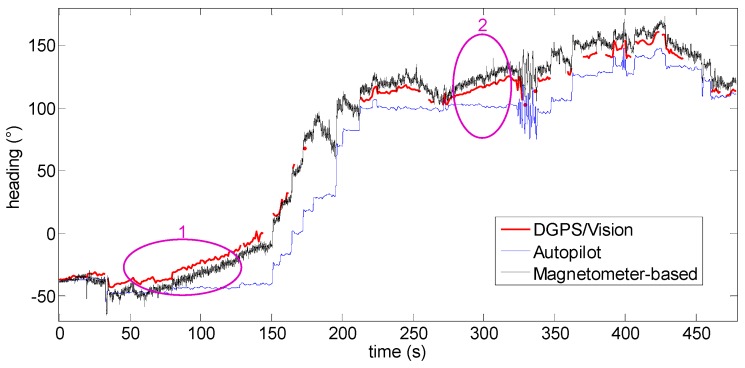
Heading as a function of time (Test 2).

**Figure 20:**
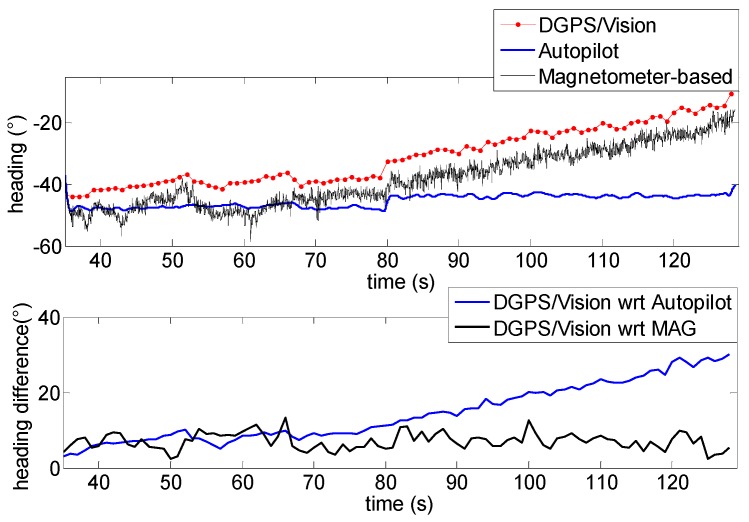
Heading angle as a function of time (**Top**), differences of DGPS/Vision with respect to autopilot and magnetometers (**Bottom**) during the first flight segment (Test 2).

Heading as a function of time is depicted in Figure 19, also in this case two flight segments are focused to point out DGPS/Vision and autopilot/magnetometer-based performance. Both flight segments are characterized by a small velocity (order of 1 m/s) and an almost constant small heading rate, of the order of 0.8°/s. Moreover, during these flight segments the baseline with deputies has been kept large enough to achieve sub-degree DGPS/Vision heading uncertainty. In these conditions, it is particularly challenging for the onboard data fusion algorithm to track heading dynamics. In fact, the first flight segment (Figure 20) shows an increasing drift of the difference between DGPS/Vision and autopilot, reaching a mean value of 14.8° (see Table 4), with the latter being almost insensitive to heading variations. On the other hand, if one neglects high frequency noise of magnetic estimates, the difference between DGPS/Vision and magnetometer-based estimates tends to be an almost constant offset of about 7.1° (Table 4). Given the similar aircraft heading, the offset is of the same order of the one experimented during test 1 (Table 3).

**Figure 21:**
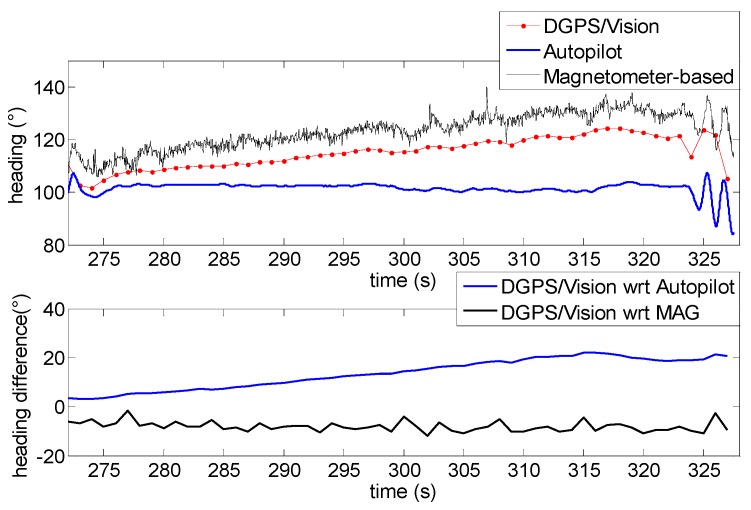
Heading angle as a function of time (**Top**), differences of DGPS/Vision with respect to autopilot and magnetometers (**Bottom**) during the second flight segment (Test 2).

From a qualitative point of view, the second flight segment (Figure 21) shows a similar behavior of DGPS/Vision and autopilot estimates, with an increasing drift of the difference that reaches a mean value of about 13.3° (Table 4), since data fusion output is relatively insensitive to very slow rotations. At a quantitative level the estimated difference is impacted by the fact that data fusion output depends on attitude dynamics history and is increasingly affected by bias instability of inertial sensors.

If one compares DGPS/Vision and magnetometer-based solutions (Table 4), as in the first flight segment an almost constant offset is obtained (removing high frequency noise of magnetic measurements). However, the estimated bias of about −8.3° is almost opposite with respect to the first flight segments in which the bias is about 7.1°. This confirms that the origin of this difference lies in the effect of uncompensated magnetometer bias and onboard magnetic fields, which are constant in the body reference frame and thus generate an effect on heading estimation error which strongly depends on quadrotor orientation within the Earth’s magnetic field.

### 6.4. Pitch and Roll

**Figure 22:**
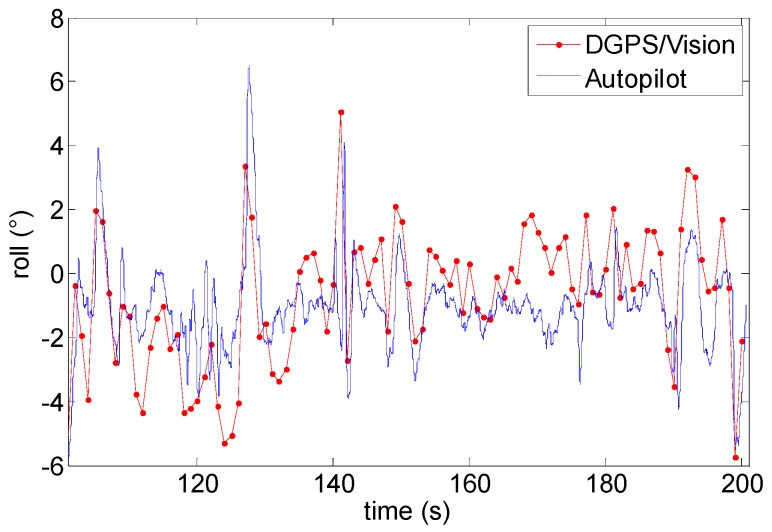
Roll angle as a function of time (Test 1).

**Figure 23:**
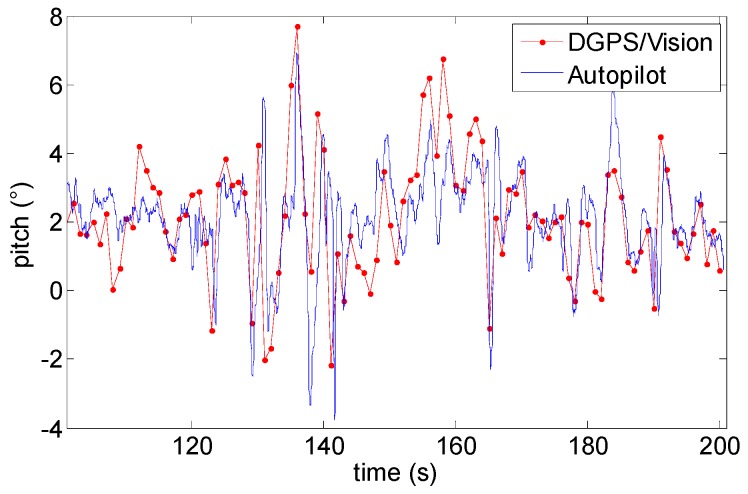
Pitch angle as a function of time (Test 1).

Although formation geometries and experimental tests have been mainly designed to optimize and analyze in detail DGPS/Vision heading angle estimation performance, for the sake of completeness it is also useful to compare pitch and roll angles as estimated by DGPS/Vision and onboard data fusion algorithm. This is done in Figure 22 and Figure 23 which are relevant to Test 1. In both cases, estimated differences fall within the accuracy levels predicted in DGPS/Vision error budgets. Due to the baseline and the formation geometry, pitch and roll accuracy levels are of the order of 0.7° and 3.5° respectively, while autopilot estimates take great advantage from gravity contribution and accelerometers measurements. However, both pitch and roll diagrams allow appreciating the good consistency between different measurements.

### 6.5. Pointing/Attitude Accuracy Results

**Figure 24:**
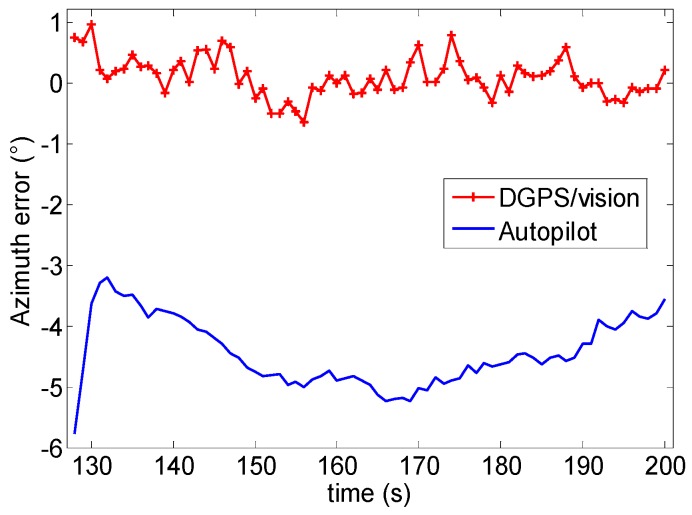
Azimuth error (°) as a function of time (Test 1-time frame 1).

**Figure 25:**
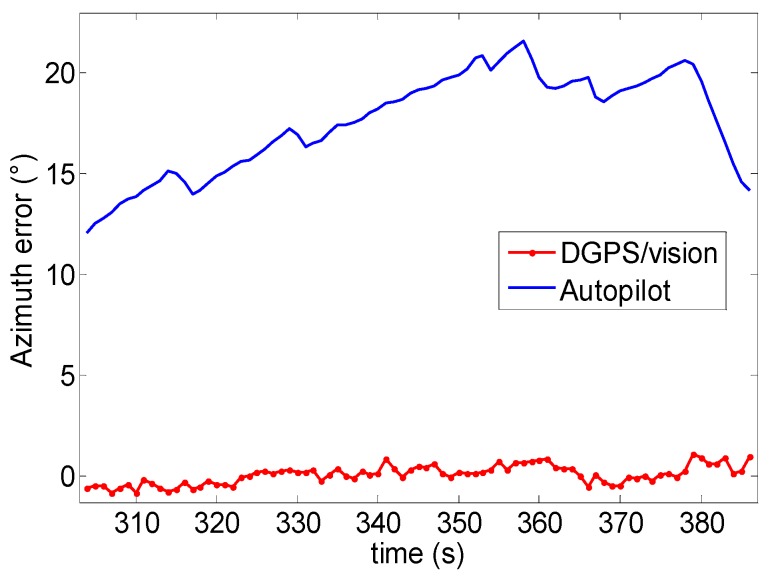
Azimuth error (°) as a function of time (Test 1-time frame 2).

Time frames 1 and 2 of Test 1 have been chosen as an example to show the accuracy achievable by using the DGPS/Vision approach, based on the strategy described in Subsection 5.2. Figure 24 and Figure 25 show the computed errors in the two time frames, both for DGPS/Vision and the Autopilot. In particular, considering the time frame 1 of Test 1 (Figure 24), the DGPS/Vision azimuth error $\Delta Az_{DGPS/Vision}$ has a mean of about 0.09° with a standard deviation of 0.3° while the autopilot azimuth error $\Delta Az_{Autopilot}$ is about 4.4° (see Table 5). Considering the time frame 2 of Test 1, (Figure 25) after the three 360° heading rotations, the $\Delta Az_{DGPS/Vision}$ mean remains of the order of 0.06° while $\Delta Az_{Autopilot}$ mean increases to about 17.6° (Table 5). These results show that, unlike the Autopilot, the DGPS/Vision errors do not show a clear dependence on flight dynamics history, and fall within the uncertainty of the reference azimuth measurements. This is consistent with [11], which shows that, depending on flight dynamics and history, the Pelican autopilot heading error can increase up to about 20°.

## 7. Conclusions

This paper presented an algorithm developed to improve UAV navigation performance in outdoor environments by exploiting cooperation among UAVs, differential GNSS and relative sensing by vision. In particular, the focus was set on attitude determination based on TRIAD algorithm.

Both numerical simulations and flight results showed the potential of sub-degree angular accuracy. In particular, proper formation geometries, and even relatively small baselines, allow achieving a heading uncertainty that can approach 0.1°, which represents a very important result taking into account typical performance levels of IMUs onboard small UAVs. Furthermore, the dependency of attitude estimation performance on formation geometry can be exploited to the navigation advantage if proper cooperative guidance laws are used to reconfigure the UAV formation as needed.

Flight experiments showed that the main factor enabling highly accurate attitude estimates is the information independence from both inertial and magnetic measurements. On the one hand, DGPS/Vision estimates are not influenced by flight history and changing inertial sensors biases, thus being insensitive to error accumulation phenomena. On the other hand, they are not affected by magnetic phenomena which are difficult to counteract in single vehicle applications since resulting errors depend on vehicle orientation.

## Figures and Tables

**Table 1:** Considered formation geometries.

	*μ*_1_ = *μ*_2_ (°)	*ε*_c_ (°)	∆*ε* (°)	L_1_ = L_2_ (m)
Case 1	0	0	[10, 90]	[50, 500]
Case 2	45	0	[10, 90]	[50, 500]
Case 3	0	45	[10, 90]	[50, 500]

**Table 2:** Pelican Navigation Sensors.

Component	Model
Gyroscopes	Analog Devices^TM^ ADXRS610
Accelerometer	Memsic^TM^ R9500
Barometer	NXP^TM^ MPXA6115A
Compass	Honeywell^TM^ HMC5843
PS Receiver	uBlox^TM^ LEA-6S

**Table 3:** Heading comparison (mean values in degrees).

Time Intervals	DGPS/Vision	Magnetometer	Autopilot Data Fusion	Difference between DGPS/Vision and	
				Magnetometer	Autopilot
*1*	−51.5	−58.8	−57.4	7.3	5.9
*2*	−48.8	−55.8	−33.3	7	−15.5
*3*	−47.5	−54.7	−45.9	7.2	−1.6

**Table 4:** Heading comparison (mean values in degrees).

Time Intervals	DGPS/Vision	Magnetometer	Autopilot Data Fusion	Difference between DGPS/Vision and	
				Magnetometer	Autopilot
*1*	−31	−38.1	−45.4	7.1	14.8
*2*	114.8	123.1	101.5	−8.3	13.3

**Table 5:** Azimuth error (°) comparison ($\Delta Az_{DGPS/Vision}$, $\Delta Az_{Autopilot}$) Test 1.

Time Intervals	$\Delta Az_{DGPS/Vision}$ Mean	$\Delta Az_{DGPS/Vision}$ Std.	$\Delta Az_{Autopilot}$ Mean	$\Delta Az_{Autopilot}$ Std.
*1*	0.09	0.3	−4.4	0.5
*2*	0.06	0.46	17.6	2.5
